# Usefulness of a novel device to divide core needle biopsy specimens in a spatially matched fashion

**DOI:** 10.1038/s41598-020-74136-3

**Published:** 2020-10-13

**Authors:** Takumi Shiraishi, Shogo Inui, Yuta Inoue, Yumiko Saito, Hideto Taga, Masatomo Kaneko, Keisuke Tsuji, Saya Ueda, Takashi Ueda, Toru Matsugasumi, Hidefumi Taniguchi, Akihisa Ueno, Takeshi Yamada, Yasuhiro Yamada, Tsuyoshi Iwata, Atsuko Fujihara, Fumiya Hongo, Osamu Ukimura

**Affiliations:** Department of Urology, Graduate School of Medical Science, Kyoto Prefectural University of Medicine, Kawaramachi-Hirokoji, Kamigyo-ku, Kyoto, 602-8566 Japan

**Keywords:** Biomedical engineering, Cancer, Diseases

## Abstract

We developed a novel dividing device that can split needle biopsy tissues along longitude axis aiming to achieve definitive molecular-biological and genetical analysis with reference of pathological diagnosis of the side-by-side divided tissue as spatially matched information. The aim of this study was to evaluate the feasibility and potential usefulness of the novel dividing device to provide the appropriate materials for molecular diagnosis. The new device was examined using mouse xenograft tumors. Real-time quantitative PCR and genetic test were performed to evaluate the feasibility and usefulness of the device. All the samples from needle biopsy were successfully divided into two pieces. Quality and quantity from divided samples harbor high enough to perform gene expression analysis (real-time PCR) and genetic test. Using two divided samples obtained from xenograft tumor model by needle biopsy, the % length of xenograft tumor (human origin) was significantly correlated with the % human genomic DNA (*p* = 0.00000608, r = 0.987), indicating that these divided samples were spatially matched. The novel longitudinally dividing device of a needle biopsy tissue was useful to provide the appropriate materials for molecular-biological and genetical analysis with reference of pathological diagnosis as spatially matched information.

## Introduction

Histopathological diagnosis has been an essential step for definitive diagnosis of cancer or disease and therapeutic decision. There are several methods to access targets for histopathological diagnosis and needle biopsy including core needle biopsy and fine-needle aspiration biopsy has been an indispensable tool to diagnose diseases because of their minimally invasiveness. In addition, surgical biopsy is sometimes applied when the access to tumors is difficult by needle biopsy and more tissues are required for its diagnosis. Furthermore, liquid biopsy targeting circulating tumor cells, circulating cell-free DNA and RNA has emerged as a promising tool to identify biomarkers in a number of cancers for personalized medicine with minimal invasiveness^1^. Of those, core needle biopsy has been one of the gold standard techniques to obtain tissues from several types of solid cancer or disease for histopathological diagnosis.

On the other hand, according to the recent significant progress of molecular biology including genetic or epigenetic analysis, the cytological feature of every pathological lesion is important to determine the most effective and suitable treatment available^2^. Therefore, it is no doubt that there is an increasing demand on obtaining appropriate samples from needle biopsy tissues for definitive molecular and genetical test as well as histopathological diagnosis^3–5^.

Several reports suggested that extensive heterogeneity between individual tumors was identified by large-scale sequencing analysis of solid cancers^6–10^. It has been also well known that cancer harbor genetic intra-tumor heterogeneity, resulting in a cause of treatment failure and drug resistance^11–19^. Therefore, it is quite important for achieving of definitive molecular analysis to take appropriate material from the targeted lesion, in which histopathological diagnosis should be defined to refer with those for the molecular analysis.

For molecular biological test, nucleic acid is usually extracted from formalin-fixed, paraffin-embedded (FFPE) tissue sections since the use of fresh frozen tissue from biopsy samples is often limited. However, purification of high-quality nucleic acid, especially RNA, from FFPE samples is often challenging. In fact, it is reported that there is wide variation between reports in terms of the accuracy of molecular analysis including genotyping and mRNA expression data produced using FFPE biospecimens^20^. Although recent study suggested a feasibility to measure the behavior of a wide variety of RNA from a single cell, such sequencer is likely too expensive to be available in common institution. Generally, additional biopsies are often required to obtain sufficient amount of tissues from needle biopsy specimens for molecular biological test. However, it is important to note that these biopsy-materials from the possibly spatially same pathological area are not guaranteed whether those are obtained from spatially matched lesion with histopathological diagnosis.

In this study, we evaluated the feasibility and usefulness of the new longitudinally dividing device of biopsy-tissue, that can divide a needle biopsy tissue into two spatially matched pieces along the longitudinal axis, enabling to analyze both histopathological diagnosis and molecular biological analysis simultaneously from a single biopsy tissue.

## Results

### Device for splitting (dividing) needle biopsY tissue in longitudinal axis (DESNY)

**Figure 1:**
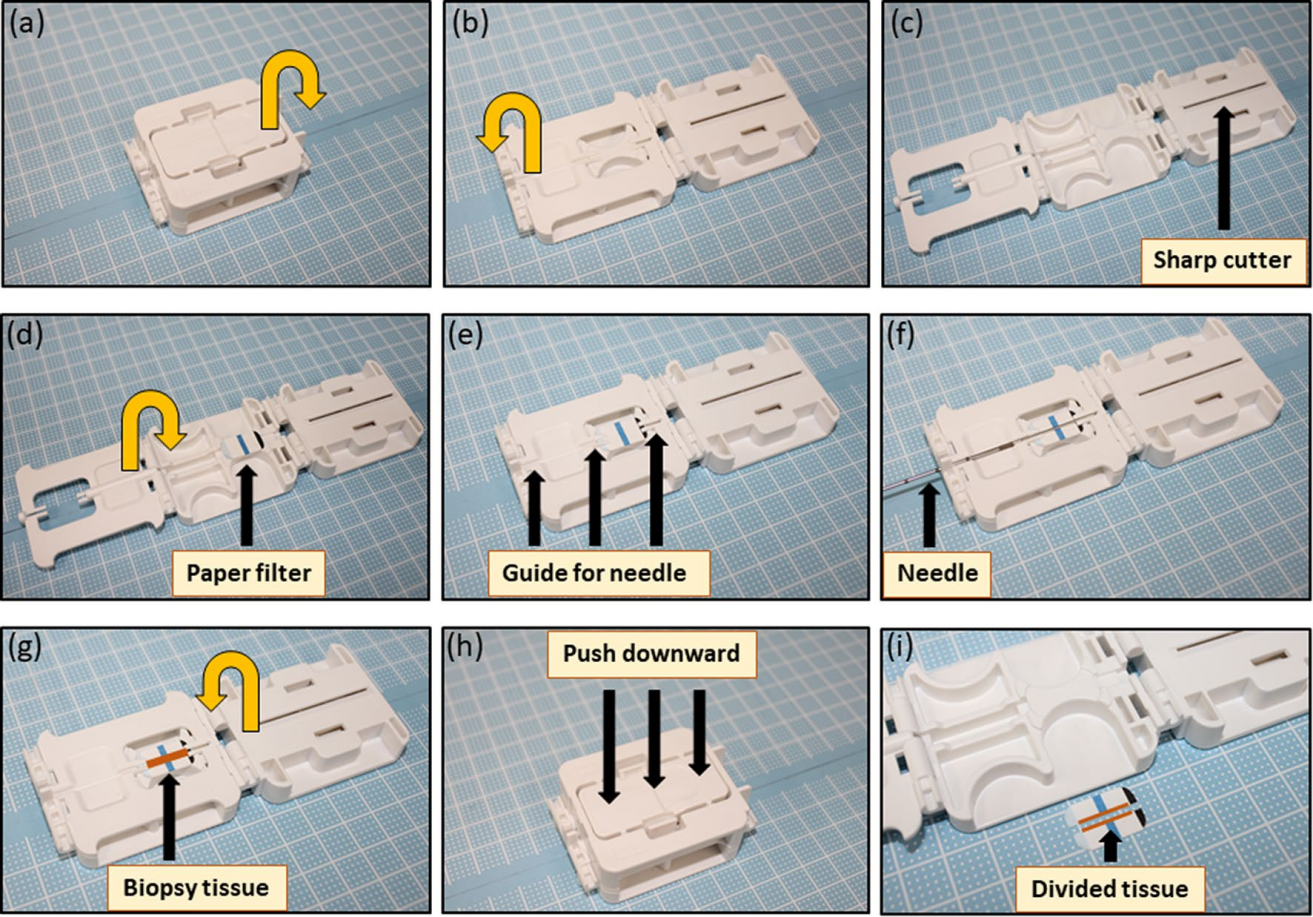
Characteristics of the new device. (**a**–**i**) There are two main characteristics including guides for a needle to place a biopsy tissue in a linear fashion on a paper and sharp cutter to divide a biopsy tissue with a filter paper in longitudinal axis.

We designed a special device that was manufactured by UMIHIRA Co., Ltd. (Kyoto, Japan). The device is only available for specimens obtained from core needle biopsy in solid tumors or tissues. The device has two main characteristics: (1) guides for a needle to place a biopsy tissue in a linear fashion, adhered on a specific filter paper, and (2) sharp cutter to divide a biopsy tissue with a filter paper in longitudinal axis, resulting in two divided tissues, adhered on a paper in a linear fashion for convenient use (Fig. 1). In results, the divided two tissues have spatially matched side-by-side information. The device used in this study is appropriate for 18G core biopsy needle which notch is within 20 mm. We confirmed that the device is available for any type of biopsy gun from different company as far as needle size and notch are same (data not shown). Furthermore, the company is preparing various types of chambers that is available for different size of needle (14G, 16G and 18G) and notch.

### Characteristics and procedure of the new device

**Figure 2:**
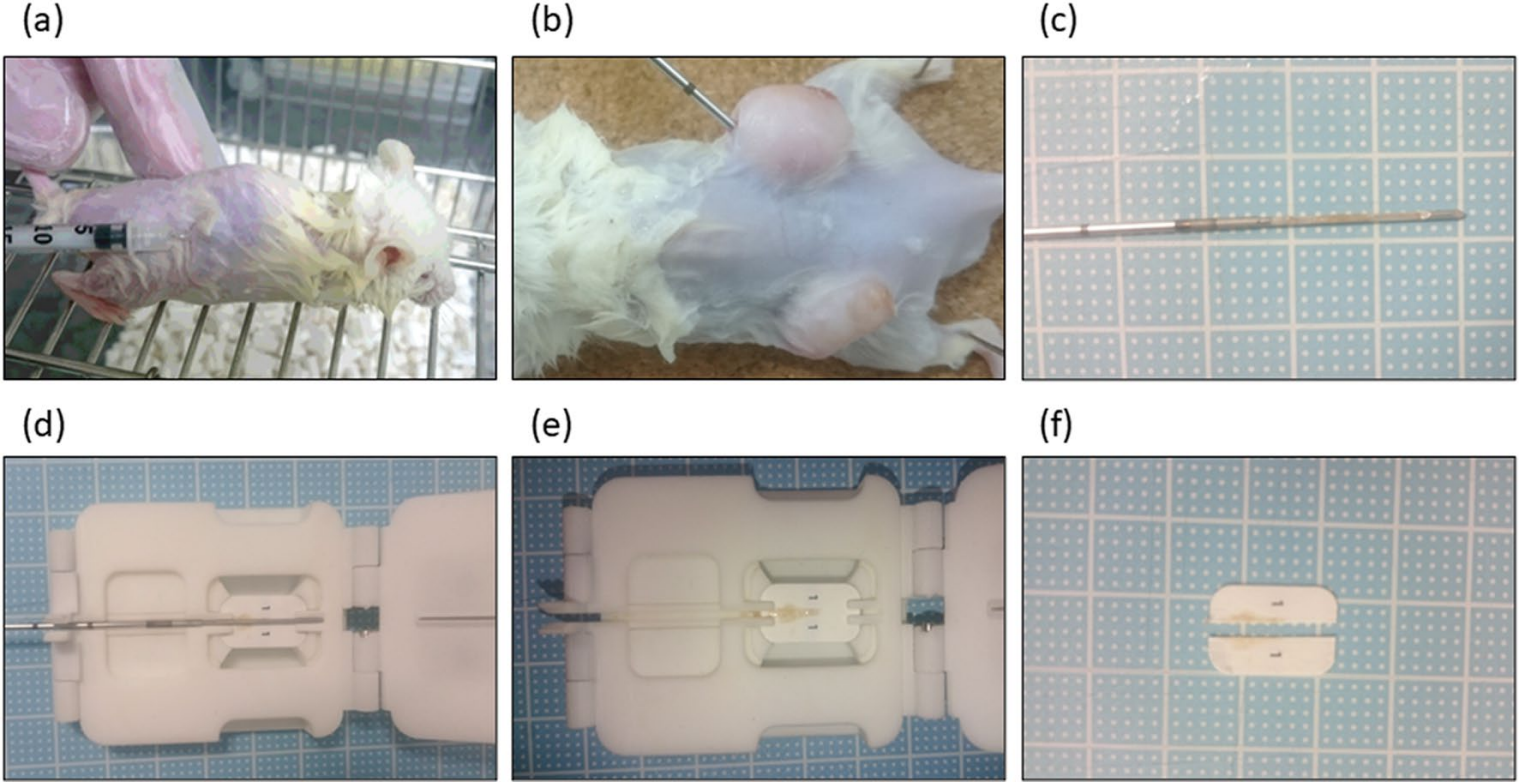
Procedure of the new device. Representative image of implanting PC3 cells into nude mice (**a**) and obtaining xenograft tumor by needle biopsy (**b**) and (**c**). (**d**–**f**) Tissue from needle biopsy is divided into two specimens in a linear fashion on a paper.

To investigate the feasibility of new device for splitting needle biopsy tissue in longitudinal axis (DESNY), we first implanted PC3 cells into nude mice and made tumor xenografts that is enough to perform core needle biopsy using 18G needle (Fig. 2a,b). The tissue obtained by needle biopsy was successfully divided into two pieces by DESNY as shown in Fig. 2. The procedure of the device was as follows; (1) expose the biopsy notch located at the distal end of the needle, (2) place the biopsy notch with the bottom up on the device with a setting paper along the needle guide, (3) carefully remove the needle leaving the tissue on the paper, (4) close the device cover and press the lid with sharp cutter downward, and (5) unfold the device and take out divided tissues from the device. One piece of the tissue was fixed by formalin and the other was immediately stored in liquid nitrogen for subsequent molecular biological analysis.

### Amount of nucleic acid from the divided specimens

DNA and RNA were extracted from the divided tissue and then quantified. The average yield of DNA and RNA were 7.4 ± 5.2 µg and 2.5 ± 1.6 µg and the mean concentration of DNA and RNA were 74.0 ± 51.6 ng/µl and 50.8 ± 29.1 ng/µl, respectively (n = 10). The amount and concentration of DNA and RNA yielded in this study were enough to use for molecular-biological and genetical analysis. For example, more than 250 ng of DNA and RNA with concentration of > 5 ng/µl and 2.5 ng/µl are required for genetic test using in this study (Oncomine Comprehensive Assay v3; Takara Bio Inc.). Furthermore, the minimum amount and concentration of DNA usually required for next-generation sequencing are more than 2 µg and 40 ng/µl for DNA.

We also compared the size of divided tissues by HE stains (n = 25). As shown in Supplemental Fig. 1, median % area of the divided samples was 46.5% and 53.5%, respectively. No statistical difference was observed between two divided groups in terms of surface area. Furthermore, we investigated the integrity of RNA in samples with and without splitting. There was no statistical difference in RNA Integrity Number (RIN) between samples with and without dividing (Supplemental Fig. 2), indicating that dividing samples by the new device did not introduce stresses on tissues in terms of RNA integrity. Moreover, as shown in Supplemental Fig. 2, damage of the cells and the architecture was not observed microscopically.

### Real-time quantitative PCR using divided specimens by DESNY

**Figure 3:**
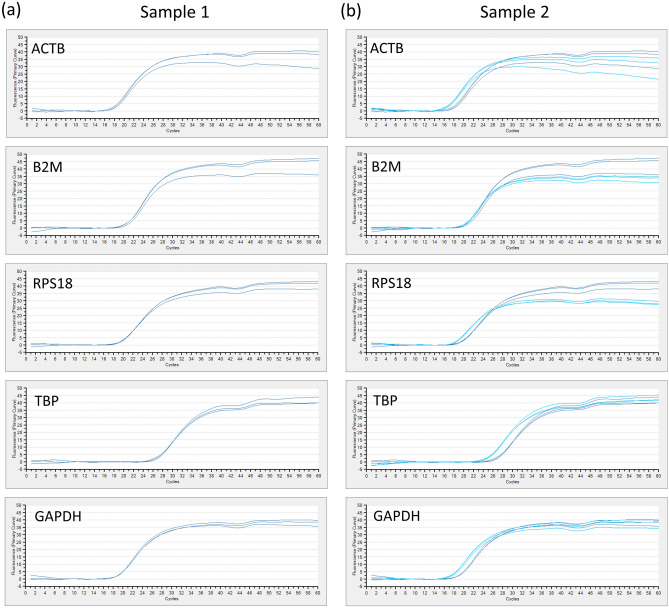
Real-time quantitative PCR using divided specimens by the new device. (**a**,**b**) The amount of ACTB, B2M, RPS18, TBP and GAPDH were analyzed by quantitative real-time PCR in two representative samples.

To see whether yielded RNA was enough to perform gene expression analysis in terms of quality and quantity, we performed Q-PCR that is a standard procedure for gene expression analysis. As shown in Fig. 3, using several primers for house-keeping genes, real-time quantitative PCR was successfully performed.

### Spatial concordance of divided specimens by DESNY

**Figure 4:**
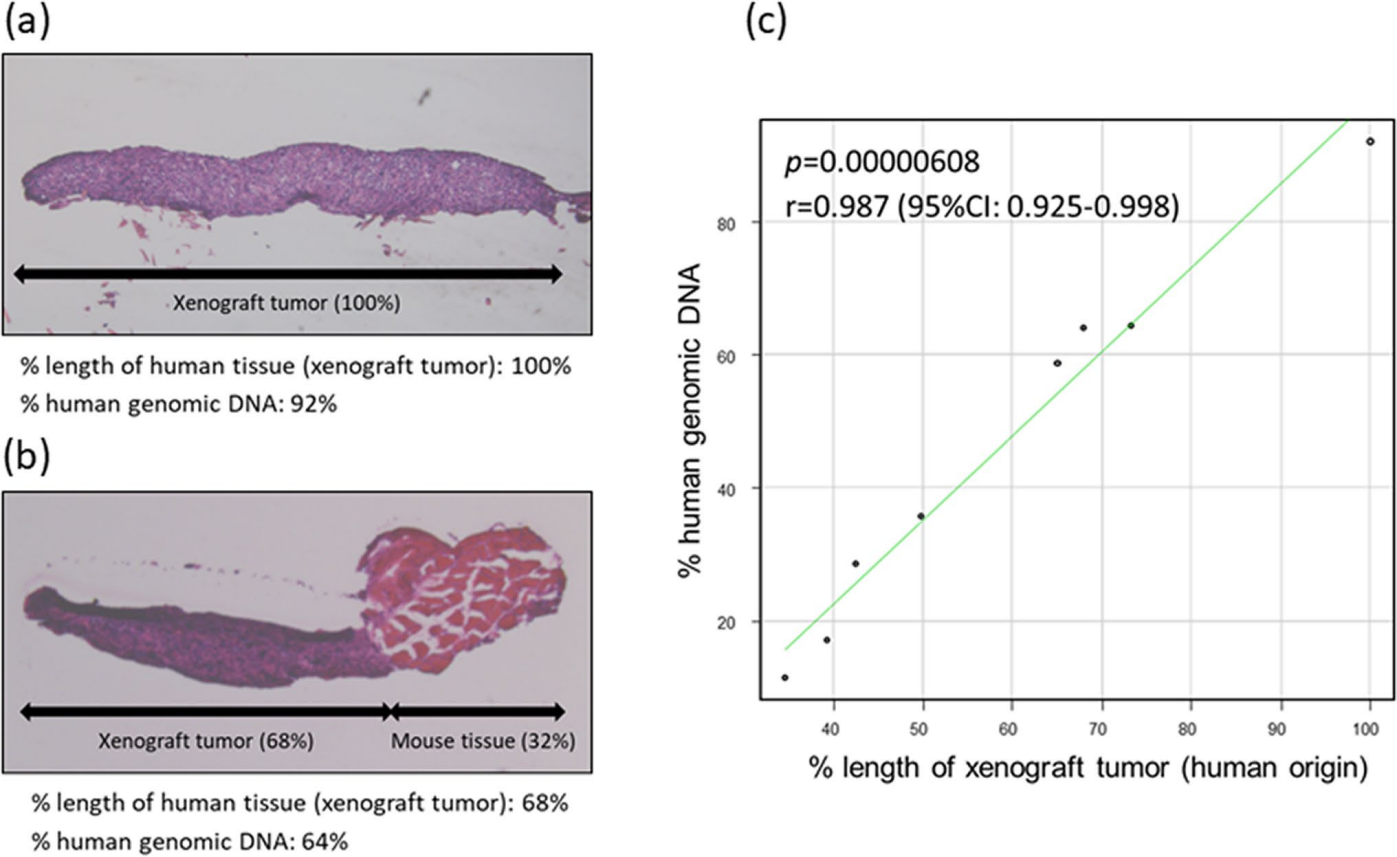
Spatial concordance of needle biopsy tissues divided by the new device. (**a**,**b**) Representative image of divided samples stained by HE. (**c**) Correlation between the % length of xenograft tumor (human origin) and the % human genomic DNA.

To verify spatial concordance of tissues from two divided specimens by DESNY, we compared one specimen for histopathological analysis (HE stains) with another specimen for molecular biological analysis (Q-PCR). In this analysis, tissues containing both mouse normal tissues and xenograft tumors (human origin) were obtained by needle biopsy. Representative results are shown in Fig. 4a,b. The % length of xenograft tumor (human origin) was significantly correlated with the % human genomic DNA (*p* = 0.00000608, r = 0.987) (Fig. 4c). The % human genomic DNA was tended to be less than the % length of xenograft tumor because of infiltration and vascularization by cells of mouse origin^21^. These results indicated that two divided specimens for histopathological and molecular biological analysis were spatially matched.

### Feasibility for genetical test using next-generation sequencing

Recently, genetic test using next-generation sequencing was accepted as a new promising method to make a treatment decision. Therefore, we finally performed the genetic test named as “Oncomine Comprehensive Assay v3” (Takara Bio Inc.). For this assay, quality and quantity of these samples were high enough to proceed the genetic test by the company quality control by fluorometric and real-time PCR determination (data not shown). In the analysis, the loss of function of p53 tumor suppressor gene was detected from xenograft tumor, which has already been reported that wild-type p53 is lost in PC3 cells^22^. The detailed results were described in Supplemental Information.

## Discussion

In the era of personalized medicine, importance of obtaining tissue-samples for molecular and genetical testing is increasing to understand tumor or disease character precisely^3,4^. On the other hand, histopathological diagnosis remains a gold standard for definitive diagnosis of cancer or disease. Core needle biopsy has been used to diagnose several types of solid cancer. However, at present, by the current core-needle-biopsy technique it is often difficult to obtain the spatially matched samples between histopathological analysis and molecular biological analysis, since the materials of 2 times core-needle-biopsies are rarely obtained from the three-dimensionally same location with the same angles. In this study, we developed new dividing device of a core-needle-biopsy-tissue that enabled to obtain two paired side-by-side samples to provide one for histopathological and another for molecular biological analysis.

We showed that the new device allowed us to obtain nucleic acid with high quality and quantity enough for both histopathological diagnosis and molecular biological analysis. Usually, nucleic acid including DNA and RNA are extracted from FFPE samples for molecular biological analysis. However, extracted nucleic acid, especially RNA, from FFPE samples often exhibited several challenging problems for molecular biological analysis in terms of quality and quantity, resulting in failure of further accurate analysis. Using the new device, the rest of divided pieces other than histopathological diagnosis can be immediately stored in liquid nitrogen, enabling samples to keep in high quality enough for later molecular biological analysis. Importantly, using this device allows that the spatial location of the targeted cells or cell-groups in the material for the molecular analysis can be confirmed with the side-by-side materials of histopathological analysis.

It is quite beneficial that samples obtained from the new device for molecular biological analysis were spatially matched tissues with histopathological specimens from needle biopsy. In a clinical setting, several times of needle biopsy are usually required when taking enough amount of tissues for molecular biological analysis in addition to histopathological analysis. However, it is important to acknowledge that these samples are not guaranteed to be the same lesion as histopathological diagnosis. Because of large extent of intra-tumor heterogeneity, a little difference of lesion obtained from needle biopsy may result in a big difference of tumor character, leading to misunderstanding of interpretation when comparing results of molecular biological analysis with those of histopathological diagnosis. Thus, the novel device reported in this study may have a potential of providing a promising therapeutic option with high accuracy by solving these problems. However, further examination using human samples in an actual clinical setting are required to demonstrate the usefulness of the new device.

The new device can be useful for several types of cancer (or disease) diagnosis and therapeutic decision. In the field of prostate cancer, for example, systematic and targeted prostate biopsy is usually conducted using 18G needle for its diagnosis, and histopathological analysis has a great influence on prostate cancer diagnosis and treatment decision. However, it has been well known that prostate cancer is an inherently multifocal disease^23^. Furthermore, multiple clonal expansions were exhibited even within a single morphologic tumor focus^24,25^. Moreover, lethal metastases have been reported to arise from a small cancer focus from an organ-confined, low-grade area of primary tumor^26^. Thus, intra-tumor heterogeneity of prostate cancer may have important consequences for personalized-medicine approaches that commonly rely on single tumor biopsy samples to portray tumor mutational landscapes. In addition to prostate cancer, the new device may propose more appropriate therapeutic decision for renal cell carcinoma (RCC) since exome sequencing of spatially separated portions of primary RCC revealed extensive intra-tumor genetic heterogeneity^27^. At present, many molecular targeting drugs and immune checkpoint inhibitors are available for the treatment of RCC. Therefore, elucidating the character of RCC precisely is quite important for selection and timing of the use of these drugs. Thus, the new device may help us to solve these current problems when providing patients with prostate and kidney cancer, for example in urology, with more precise personalized medicine.

As disadvantage of the new device, it is important for users to acknowledge a possibility that the sample adequacy is potentially compromised for pathological and molecular assessment by reducing sample size since the adequacy of tumor sampling remains a critical issue in treatment planning^28^. In general, the capability of needle biopsy itself to provide samples adequate for diagnostic and prognostic analysis remains limited^29^. Thus, there has always been a debate between the risk and benefits of biopsy versus surgical excision in oncology with reference to the adequacy of diagnosis, prognostic accuracy, and suitability for evaluation of predictive biomarkers^30^. In this context, uncertainty of histopathological diagnosis by reducing the volume of samples when using the new device might be a problem especially when the main purpose of biopsy is to investigate whether a tumor is malignant or benign. Furthermore, if the treatment of disease is well established and effective, the additional information from molecular and genetic assessment by using the new device might be limited. Thus, usefulness of the device highly depends on the indication or objective of biopsy in which patients take a core needle biopsy. Therefore, in the actual clinical setting, physician should use the new device after understanding the balance between disadvantage introduced by reducing sample size and possible advantage of obtaining information from molecular and genetic analysis.

Although we showed that there was no statistical difference between two divided samples in terms of surface area, it is true that samples could not always be divided into two equal parts completely. Therefore, in the actual clinical setting, we propose the following scenario. For example, when 3 biopsies are performed for the same target, one is for histopathological analysis without dividing and the rest of other two specimens are divided. In one divided paired sample, the potentially bigger sample is selected to molecular assessment. In another divided paired samples, the potentially bigger sample is selected to histopathological assessment. This could provide balanced information of both histopathological and molecular analysis with minimizing the disadvantage of possible reduced sample volume by dividing. Thus, physicians would determine which samples should be submitted to histopathological or molecular assessment considering various actual clinical situations, such as indication or objective of the biopsy.

## Conclusion

The novel longitudinally dividing device of needle-biopsy-tissue was useful to provide the appropriate paired materials, in which one is for molecular-biological analysis including genetic (DNA, or RNA) test and another is for pathological diagnosis of the side-by-side divided tissue with the molecular-biological analysis. Using this device, the spatial location of the targeted cells or cell-groups in the material for the molecular analysis can be confirmed with the side-by-side materials of histopathological analysis.

Since it is important to perform a molecular-biological and genetical analysis with reference of the histopathological diagnosis using the spatially matched materials, the device would become a promising tool to provide a significant information to offer personalized medicine.

## Materials and methods

### Cells and mouse xenograft tumor model

The human prostate cancer cell lines, PC3, was maintained in RPMI-1640 medium with 10% fetal bovine serum (10% FBS) at 37 °C in a humidified atmosphere containing 5% CO_2_. Five million PC3 cells was suspended in 200 µl of media and subcutaneously injected into the bilateral flank of 6-week-old SCID mice or nude mice. The size of tumors was measured with a micrometer caliper every week, and xenograft tumors and normal mouse tissues were obtained after tumor volumes were became large (8.3 ± 1.3 mm long and 7.1 ± 1.1 mm wide) enough to perform core needle biopsy using 18G needle. All the experiments were conducted under general anesthesia. These experiments were conducted in accordance with the Declaration of Helsinki and approved by the Kyoto Prefectural University of Medicine Institutional Animal Care and Use Committee (M29-564).

### RNA/DNA extraction and quantitative real-time PCR

Total RNA and DNA was isolated using RNeasy mini kit and DNeasy mini kit, respectively according to the manufacture’s protocol (Qiagen, Valencia, CA, USA). First strand cDNA was made from 0.5 µg RNA using PrimeScript RT Master Mix (Takara Bio Inc., Shiga, Japan) following the manufacturer’s protocol in a total volume of 10 µL and suspended in distilled water up to 100 µl. Quantitative real-time PCR (Q-PCR) was carried out using 2 µl of cDNA template by Thermal Cycler Dice Real Time System (Takara Bio Inc., Shiga, Japan). Housekeeping Gene Primer Set (Takara Bio Inc., Shiga, Japan) was used as PCR primers for Q-PCR.

### Quantitative analysis of DNA derived from human and mouse cells

Mouse Feeder Cell Quantification Kit (Takara Bio Inc., Shiga Japan) was used to calculate a content ratio of different variety in needle biopsy tissues obtained from mouse xenograft tumor according to the manufactured protocol. Briefly, the amount of mouse and human genomic DNA in a divided sample was assessed by Q-PCR using the kit containing primer sets specific for mouse and human genomic DNA. The formula to calculate % human genomic DNA was as follows; % human genomic DNA = human genomic DNA/total DNA (mouse + human) × 100.

### Statistical analysis

Correlation coefficient was analyzed by Pearson's test. All statistical analyses were performed with EZR (Saitama Medical Center, Jichi Medical University, Saitama, Japan), which is a graphical user interface for R (The R foundation for Statistical Computing, Vienna, Austria). More precisely, it is a modified version of R commander designed to add statistical functions frequently used in biostatistics^31^. *P* values less than 0.05 were considered to be statistically significant.

## Supplementary information

Supplementary Information

Supplementary Table
