# Supplementary material for: Usefulness of a novel device to divide core needle biopsy specimens in a spatially matched fashion
Source: Sci Rep. 2020 Oct 13;10:17098. doi: 10.1038/s41598-020-74136-3 (PMC7555856; doi:10.1038/s41598-020-74136-3)
Supplement: Supplementary file 1 — Supplementary Information [file 41598_2020_74136_MOESM1_ESM.docx]

**Supplementary Material**

**Title:** Usefulness of a novel device to divide core needle biopsy specimens in a spatially matched fashion

**Author:** Takumi Shiraishi, Shogo Inui, Yuta Inoue, Yumiko Saito, Hideto Taga, Masatomo Kaneko, Keisuke Tsuji, Saya Ueda, Takashi Ueda, Toru Matsugasumi, Hidefumi Taniguchi, Akihisa Ueno, Takeshi Yamada, Yasuhiro Yamada, Tsuyoshi Iwata, Atsuko Fujihara, Fumiya Hongo, Osamu Ukimura

**Institution:** Department of Urology, Kyoto Prefectural University of Medicine, Kyoto, Japan

**Address:** Kawaramachi-Hirokoji, Kamigyo-ku, Kyoto 602-8566, Japan

TEL: +81-75-251-5595

FAX: +81-75-251-5598

E-mail: [ukimura@koto.kpu-m.ac.jp](mailto:ukimura@koto.kpu-m.ac.jp)

**Correspondence:** Osamu Ukimura, M.D., Ph.D.

Department of Urology, Graduate School of Medical Science, Kyoto Prefectural University of Medicine, Kawaramachi-Hirokoji, Kamigyo-ku, Kyoto 602-8566, Japan

TEL: +81-75-251-5595

FAX: +81-75-251-5598

E-mail: ukimura@koto.kpu-m.ac.jp

**Supplemental Materials and Methods**

**Calculations of surface area of divided samples by the new device**

Samples from beef were divided by the new device (DESNY) and stained by HE. Percent (%) surface area is analyzed by ImageJ software (http://imagej.nih.gov/ij/index.html) with Manual Tracking.

**Measurement of RNA Integrity Number (RIN)**

Samples were obtained from chicken liver. Total RNA was isolated using RNeasy mini kit according to the manufacture’s protocol (Qiagen, Valencia, CA, USA). Total RNA (3ug) extracted from samples with and without dividing was electrophoresed with 1% agarose-TAE gel and stained with ethidium bromide. Integrity of RNA was analyzed by using Bioanalyzer (Agilent Technologies, CA, USA).

**Legend for Supplemental Information**

The results of genetic test named as “Oncomine Comprehensive Assay v3” (Takara Bio Inc.) using samples obtained by the novel longitudinally dividing device of needle-biopsy-tissue.

**Supplemental Figures**


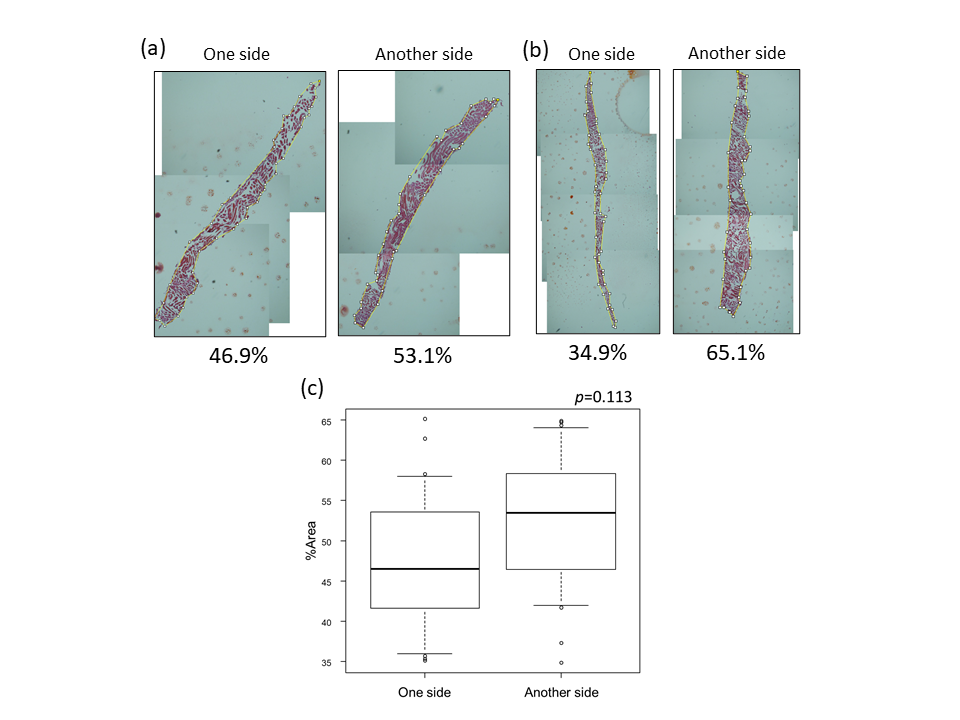


**Supplemental Figure 1. Percent area of divided samples by the new device**

(a) and (b) Representative image of two divided samples stained by HE. Percent (%) surface area is analyzed by ImageJ software with Manual Tracking. (c) Box plot of percent (%) area between two divided samples by the new device. The middle region of each box plot (box body) covers 50% of the individuals, and the region between the upper and the lower transverse lines covers 90% of the % area, whereas the points outside of the box represent outlier.


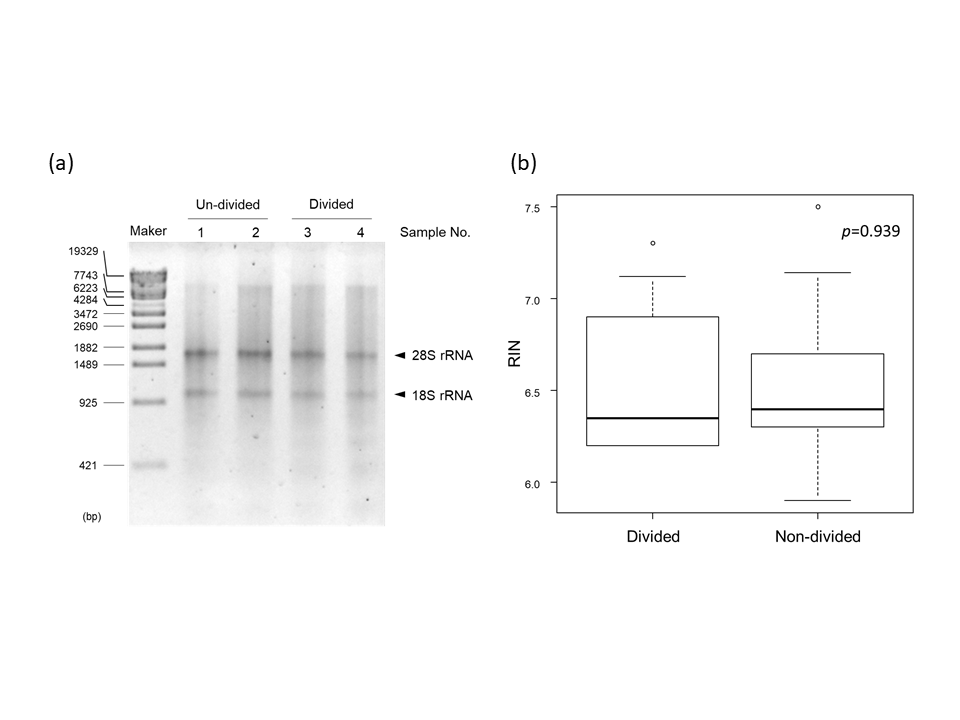


**Supplemental Figure 2. Percent area of divided samples by the new device**

(a) Representative image of samples with and without dividing electrophoresed with 1% agarose-TAE gel. (b) Box plot of RNA Integrity Number between non-divided and divided samples by the new device. The middle region of each box plot (box body) covers 50% of the individuals, and the region between the upper and the lower transverse lines covers 90% of the % RNA Integrity Number, whereas the points outside of the box represent outlier.
